# SpeB of *Streptococcus pyogenes* Differentially Modulates Antibacterial and Receptor Activating Properties of Human Chemokines

**DOI:** 10.1371/journal.pone.0004769

**Published:** 2009-03-10

**Authors:** Arne Egesten, Anders I. Olin, Helena M. Linge, Manisha Yadav, Matthias Mörgelin, Anna Karlsson, Mattias Collin

**Affiliations:** 1 Division of Respiratory Medicine, Department of Clinical Sciences Lund, Lund University, Lund, Sweden; 2 Division of Infection Medicine, Department of Clinical Sciences Lund, Lund University, Lund, Sweden; 3 Department of Rheumatology and Inflammation Research, University of Göteborg, Göteborg, Sweden; Columbia University, United States of America

## Abstract

**Background:**

CXC chemokines are induced by inflammatory stimuli in epithelial cells and some, like MIG/CXCL9, IP–10/CXCL10 and I–TAC/CXCL11, are antibacterial for *Streptococcus pyogenes*.

**Methodology/Principal Findings:**

SpeB from *S. pyogenes* degrades a wide range of chemokines (i.e. IP10/CXCL10, I-TAC/CXCL11, PF4/CXCL4, GROα/CXCL1, GROβ/CXCL2, GROγ/CXCL3, ENA78/CXCL5, GCP-2/CXCL6, NAP-2/CXCL7, SDF-1/CXCL12, BCA-1/CXCL13, BRAK/CXCL14, SRPSOX/CXCL16, MIP-3α/CCL20, Lymphotactin/XCL1, and Fractalkine/CX3CL1), has no activity on IL-8/CXCL8 and RANTES/CCL5, partly degrades SRPSOX/CXCL16 and MIP-3α/CCL20, and releases a 6 kDa CXCL9 fragment. CXCL10 and CXCL11 loose receptor activating and antibacterial activities, while the CXCL9 fragment does not activate the receptor CXCR3 but retains its antibacterial activity.

**Conclusions/Significance:**

SpeB destroys most of the signaling and antibacterial properties of chemokines expressed by an inflamed epithelium. The exception is CXCL9 that preserves its antibacterial activity after hydrolysis, emphasizing its role as a major antimicrobial on inflamed epithelium.

## Introduction

*Streptococcus pyogenes* is a strictly human pathogen that preferentially colonizes the pharynx and the skin. Bacteria disseminating from the primary site may cause life-threatening sepsis, necrotizing fasciitis, and a toxic shock syndrome [1], [2], [3]. Despite the potential virulence of *S. pyogenes*, many individuals are healthy carriers of the bacteria in their upper airways, demonstrating that the bacteria can colonize epithelial surfaces without eliciting an inflammatory response.

Extracellular enzymes from *Streptococcus pyogenes* have been extensively studied and shown to be of importance for the pathogenesis of this human pathogen. One of the most studied enzymes is the streptococcal cysteine proteinase, SpeB. Several *in vitro*, *in vivo*, and clinical studies have suggested a role for SpeB as an important virulence factor [4], [5], [6], [7], [8]. The role for SpeB as virulence factor in mouse models is not uncontroversial and there have been reports using SpeB deletion mutants indicating that SpeB is not a virulence factor [9], [10]. SpeB has the ability to degrade the human extracellular matrix protein fibronectin and vitronectin, release inflammatory mediators such as interleukin 1β and bradykinin from their precursors, cleave or degrade immunoglobulins and complement factors, and also bind to the human cell surface receptors integrins [11], [12], [13], [14], [15], [16], [17], [18], [19], [20], [21].

Dendritic cells, macrophages, and T-cells that reside in sub-epithelial tissues recognize bacterial antigens, resulting in the production of T-helper 1 polarized pro-inflammatory cytokines, including IFN–γ and TNF–α. These cytokines cause an inflamed phenotype of epithelial cells, resulting in the production of host defense molecules, including chemokines [22], [23]. Chemokines are divided into four groups, XC, CC, CXC, and CX_3_C chemokines, depending on the presence of conserved NH_2_-terminal cysteine residues [24]. MIG/CXCL9, IP–10/CXCL10 and I–TAC/CXCL11 belong to the group of CXC chemokines [25], [26], [27]. They all share the ability to signal through CXC chemokine receptor 3 (CXCR3), which is present on T cells and natural killer cells. Ligand binding to the receptor results in the activation and recruitment of these cells to sites of inflammation [28], [29]. In addition to the CXCR3 interactions, CXCL9, CXCL10, and CXCL11 also possess direct antibacterial activity *in vitro* [30]. Several other chemokines that are investigated in the present study also possess direct antimicrobial activity (Table 1).

**Table 1:** Known antimicrobial activities of the chemokines used in the present study.

Chemokine	Activity	Reference(s)
CXCL1/GRO-α	*E. coli*, *S. aureus*, *S. typhimurium*, *C. albicans*	[59], [60]
CXCL2/GRO-β	*E. coli*, *S. aureus*	[59]
CXCL3/GRO-γ	*E. coli*, *S. aureus*	[59]
CXCL4/PF4	*S. typhimurium*	[60], [61]
CXCL5/ENA-78	*S. pyogenes*	[62]
CXCL6/GCP-2	*S. pyogenes*, *E. coli*, *P. aeruginosa*, *S. dysgalactiae*, *S. aureus*, *N. gonorrhoeae*, *E. faecalis* / No activity on *E. coli*, *S. aureus*	[62], [63]/[59]
CXCL7/NAP-2	*S. pyogenes*	[62]
CXCL8/IL-8	*S. aureus*, *S. typhimurium*, *C. albicans*	[60]
CXCL9/MIG	*E. coli*, *S. aureus*, *S. pyogenes*, *N. gonorrhoeae*	[31], [59], [64]
CXCL10/IP10	*E. coli*, *S. aureus*, *S. pyogenes*	[31], [59]
CXCL11/I-TAC	*E. coli*, *S. aureus*, *S. pyogenes*	[31], [59]
CXCL12/SDF-1α	*E. coli*, *S. aureus*	[59]
CXCL13/BCA-1	*E. coli*, *S. aureus*	[59]
CXCL14/BRAK	*E. coli*, *S. aureus*	[59]
CXCL16/SRPSOX	N/D	-
CCL5/RANTES	No activity on *E. coli*, *S. aureus / S. aureus*, *S. typhimurium*, *C. albicans*	[59] / [60]
CCL20/MIP-3α	*E. coli*, *S. aureus*	[59]
CCL28/MEC	*E. coli*, *S. aureus*, *S. pyogenes*, *S. mutans*, *K. pneumoniae*, *P. aeruginosa*, *C. albicans*	[65]
XCL1/Lymphotactin	*E. coli*, *S. aureus*, *S. typhimurium*, *C. albicans*	[59], [60]
CX3CL/Fractalkine	No activity on *E. coli*, *S. aureus*	[59]

In a recent study, we showed that CXCL9, CXCL10, and CXCL11 kill *S. pyogenes*, and that in particular CXCL9, is produced at bactericidal concentration by inflamed pharyngeal cells both *in vivo* and *in vitro* [31]. Furthermore, one of the major surface proteins of *S. pyogenes*, the M protein, induces increased production of MIG in IFN–γ stimulated pharyngeal cells [32].

Proteolytic processing can significantly alter the signaling activity of chemokines and is most likely one of the major regulatory mechanisms for chemokines. The host proteases gelatinase B/matrix metalloproteinase-9 (MMP-9) and neutrophil collagenase/MMP-8 process several CXC chemokines including CXCL9 and CXCL10 [33], [34]. MMP-9 degrades CXCL10 and cleaves CXCL9 at three different sites in its extended carboxy-terminal region, while MMP-8 degrades CXCL9 and cleaves CXCL10 at two positions [34]. It is currently not known how proteolytic processing influences the antibacterial activity of CXCL9, CXCL10, and CXCL11.

Corruption of innate immunity by bacterial proteases is a rapidly growing field of research (for a recent comprehensive review see [35]), and processing of chemokines by bacterial proteases has recently gained substantial attention. Metalloproteases from *Pseudomonas aeruginosa* degrade several chemokines [36]. Furthermore, an elastase from this bacterium degrades the chemokine-like antimicrobial peptide LL-37 [37]. In the same study by Schmidtchen *et al.* SpeB from *S. pyogenes* was also shown to degrade LL-37, which is of particular interest for the present study. *S. pyogenes* has recently been shown to produce another interesting protease, the cell wall-anchored serine protease, SpyCEP, that degrades the CXC chemokine, IL-8/CXCL8, and thus promotes resistance against of neutrophil killing [38], [39], [40], [41]. SpyCEP was very recently also shown to degrade two additional CXC chemokines, GCP-2/CXCL6 and GROα/CXCL1 resulting in impaired neutrophil recruitment [42]. These findings, together with previous observations concerning direct and indirect enzymatic activities on host immune factors, highlight *S. pyogenes* as one of the most versatile modulators of innate and adaptive immune responses.

## Results

### SpeB degrades or cleaves most human chemokines, but not CXCL8 and CCL5

**Figure 1:**
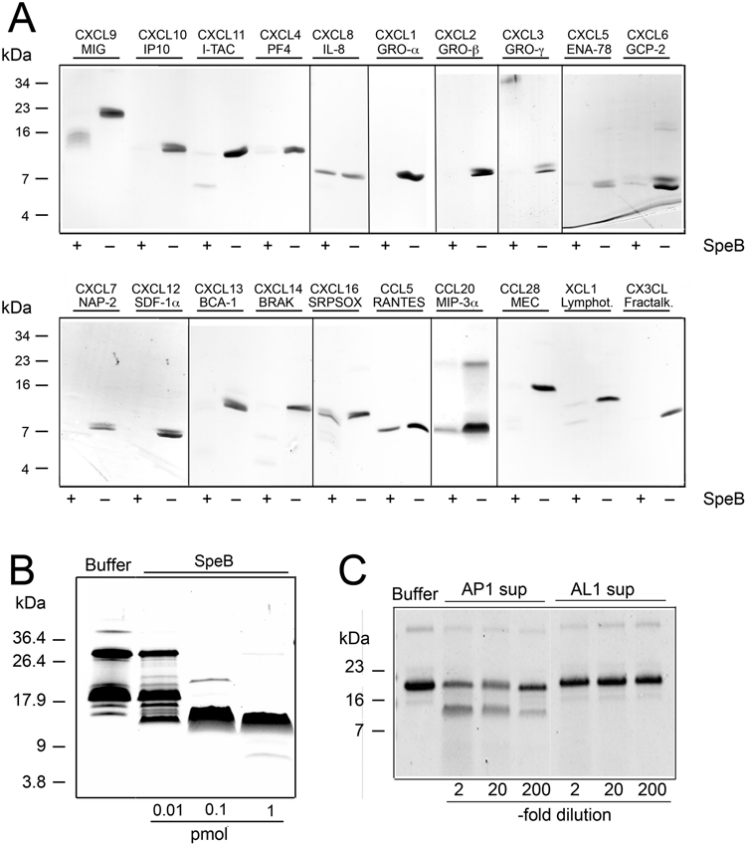
SpeB degrades or processes several human chemokines. Panel A: The streptococcal cysteine proteinase was incubated with human recombinant chemokines and separated on 16.5% Tris-Tricine SDS-PAGE. Chemokines (standardized ligand and common name) are indicated above the panels and presence or absence of SpeB during incubation is indicated with minus or plus signs below the panels. Panel B: Recombinant MIG/CXCL9 was incubated with 1, 0.1, or 1 pmol of active SpeB and separated on 16.5% Tris-Tricine SDS-PAGE. CXCL9 incubated with buffer alone is shown to the left. Panel C: Two micrograms of recombinant human CXCL9 was incubated with dilutions (as indicated) of sterile filtered culture supernatants from wild type *S. pyogenes* AP1 and SpeB-negative isogenic derivative AL1 and separated on 16.5% Tris-Tricine SDS-PAGE. CXCL9 incubated with buffer alone is shown to the left.

Since it has been shown that the streptococcal cysteine proteinase SpeB can cleave and inactivate the antimicrobial peptide LL-37 [37], we hypothesized that SpeB also could degrade or process chemokines that share many properties with antimicrobial peptides. We therefore incubated 2 µg of the human chemokines CXCL9, CXCL10, CXCL11, CXCL4, CXCL8, CXCL1, CXCL2, CXCL3, CXCL5, CXCL6, CXCL7, CXCL12, CXCL13, CXCL14, CXCL16, CCL5, CCL20, CCL28, XCL1, and CX3CL1 with a small amount of SpeB (pmol range) for 2 hours. When the samples were analyzed by SDS-PAGE, this revealed that SpeB partially or completely degrades most of these chemokines (Fig. 1A). However, CCL5 and CXCL8 were completely resistant to cleavage by SpeB, and CXCL16 and CCL20 were only partly degraded. It should be added for clarity that both intact and fragmented chemokines sometimes appear as multiple bands due to oligomerization, and that CXCL9 migrates on SDS-PAGE as an apparently larger molecule than its actual mass.

What was most interesting in relation *S. pyogenes* is that there were differential SpeB activities on the closely related ELR-negative CXC-chemokines CXCL9, CXCL10, and CXCL11, where CXCL9 was processed to a smaller fragment rather than being degraded, CXCL10 was almost completely degraded, and CXCL11 was completely degraded. Furthermore, SpeB processes CXCL9 at lower concentrations and the generated fragment is resistant to further degradation even after prolonged incubation or higher concentrations of SpeB (Fig. 1B and data not shown). It should be noted that in all SDS-PAGE separations except in Fig. 1B, we utilized an SDS-PAGE loading buffer containing dithiothreitol (DTT) and iodacetamide to disrupt oligomers of chemokines. In the experiments shown in Fig. 1B we used standard SDS-PAGE loading buffer only containing β-mercaptoethanol as a reducing agent. This allows for the visualization of oligomers, and therefore our interpretation of Fig. 1B is that SpeB either aids in the disruption of CXCL9 oligomers, or has direct proteolytic activity on both monomers and oligomers of CXCL9. Furthermore, CXCL9 was partly processed to fragments, comparable to what could be seen with purified SpeB, after incubation with sterile filtered culture supernatant from wild type AP1 bacteria, while supernatant from the isogenic SpeB negative strain did not hydrolyze CXCL9 (Fig 1C). This indicates that under these conditions there is no other secreted factor than SpeB from *S. pyogenes* that can degrade CXCL9.

The three CXC chemokines CXCL9, 10, and 11 are closely related, act through the same receptor (CXCR3), are expressed in the context of streptococcal pharyngitis, are antibacterial towards *S. pyogenes* [31], and are induced by the cell wall-anchored M protein from *S. pyogenes* [32]. We therefore decided to further elucidate the receptor activating and antimicrobial activity of CXCL9, CXCL10, and CXCL11 after SpeB hydrolysis.

### CXCL9, but not CXCL10 and CXCL11 remains antibacterial after SpeB hydrolysis

**Figure 2:**
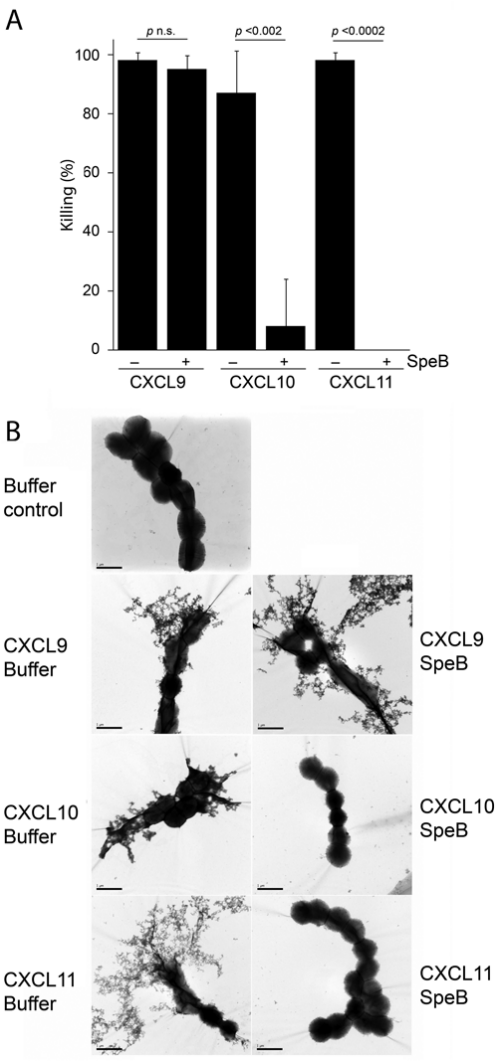
The effect of SpeB on the antibacterial activity of 0.5 µM CXCL9, CXCL1010, and CXCL11. Panel A: CXCL9, CXCL10, and CXCL11 incubated with SpeB were compared to the intact molecules for killing of *S. pyogenes* strain AP1. Bars represent mean bacterial killing in percent with standard deviation determined from at least three independent experiments. Panel B: Transmission electron micrographs of AP1 bacteria incubated with intact (CXCL9-11, Buffer) or SpeB treated (CXCL9-11, SpeB) CXCL9, CXCL10, and CXCL11. Control bacteria were incubated with buffer alone. Scale bar within each picture represents 1 µm.

In particular CXCL9, and to some degree CXCL10, CXCL11, seem to be important for the antibacterial activity against *S. pyogenes* on inflamed respiratory tract epithelium [31]. We therefore investigated how SpeB cleavage affected the antimicrobial activity of these three chemokines on *S. pyogenes* strain AP1 of the important M1 serotype. A viable count assay revealed that, not surprisingly, the completely degraded CXCL11 and the nearly completely degraded CXCL10 had very low or no killing activity towards *S. pyogenes* AP1 bacteria, while the corresponding intact CXCL10 and CXCL11 very efficiently killed the bacteria (Fig. 2B, CXCL10 and CXCL11±SpeB). This was also confirmed by transmission electron microscopy (TEM) of bacteria incubated with intact and SpeB-treated CXCL10 and CXCL11, where both CXCL10 and CXCL11 caused membrane disruption and disintegration of bacterial chains while SpeB-treated CXCL1010 and CXCL11 had no visible effect on that bacteria when compared to bacteria incubated with buffer alone (Fig. 2B, Control, CXCL10 and CXCL11, Buffer / SpeB). In contrast to the findings for CXCL10 and CXCL11, there was no significant difference in killing of AP1 bacteria between SpeB-treated and intact CXCL9 (Fig. 2A, CXCL9±SpeB). This was also confirmed by TEM where CXCL9 irrespective of SpeB-treatment caused membrane disruption and disintegration of bacterial chains (Fig 2B, CXCL9, Buffer / SpeB).

Taken together, these experiments indicate that SpeB destroys the antibacterial activity of CXCL10 and CXCL11, while SpeB-processing of CXCL9 with loss of nearly half of the molecule does not influence the potent antibacterial activity of CXCL9 against *S. pyogenes*.

### SpeB-cleaved CXCL9 does not signal through CXCR3

**Figure 3:**
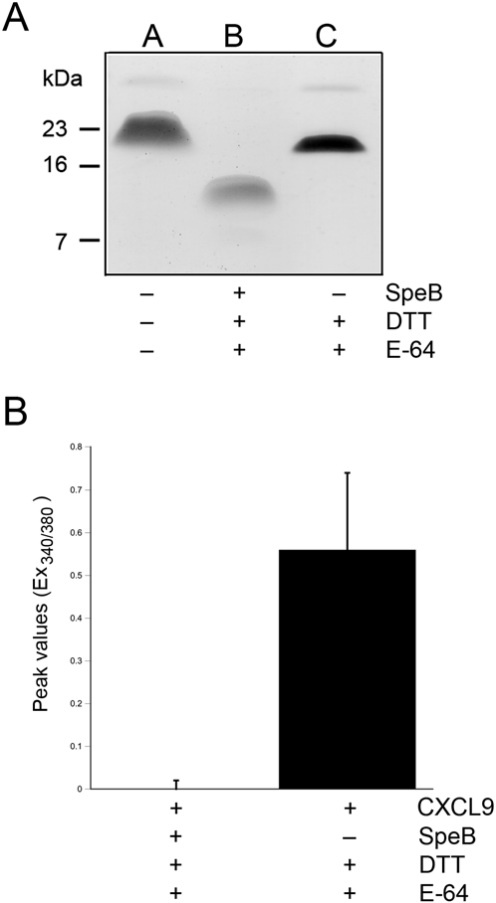
SpeB-hydrolyzed CXCL9 does not signal through CXCR3. Panel A: CXCL9 was incubated with SpeB / DTT or DTT and terminated by addition of the cysteine proteinase inhibitor E-64. Samples were separate on 16.5% SDS-PAGE and compared with intact CXCL9. Panel B: Analysis of calcium flux in Fura-2 loaded CXCR3-expressing pre-B cells upon stimulation with MIG incubated with SpeB / DTT or CXCL9 incubated with DTT alone, both terminated with E-64. Peak values of the Ex340/380 ratio fluorescence curves are presented as means and standard deviations from three independent experiments.

Since the SpeB-generated CXCL9 fragment had retained antibacterial activity, we decided to examine if the receptor activating properties were affected. In order to do this, we incubated CXCL9 under reducing conditions (DTT) with or without SpeB. To minimize the proteolytic activity SpeB might have on cells, we terminated the reactions using the specific cysteine proteinase inhibitor E-64 [43]. These samples were analyzed by Tris-Tricine SDS-PAGE revealing that DTT and E-64 treated CXCL migrated as an apparently slightly smaller protein than untreated CXCL9 (Fig. 3A, lanes A and C). This could possibly be explained by some minor spontaneous hydrolysis of CXCL9. In contrast, addition of SpeB to the reaction led to a complete conversion of CXCL9 into a fragment with an apparent mass of approx. 10 kDa (Fig. 3A, lane B). Intact and SpeB-treated CXCL9 was then investigated for receptor interaction by measuring their effects on intracellular calcium levels in CXCR3-transfected pre-B cells. The results show that SpeB-processed CXCL9 had lost all the receptor-stimulating capacity of intact CXCL9.

### Identification and purification of the SpeB-generated fragment of CXCL9

**Figure 4:**
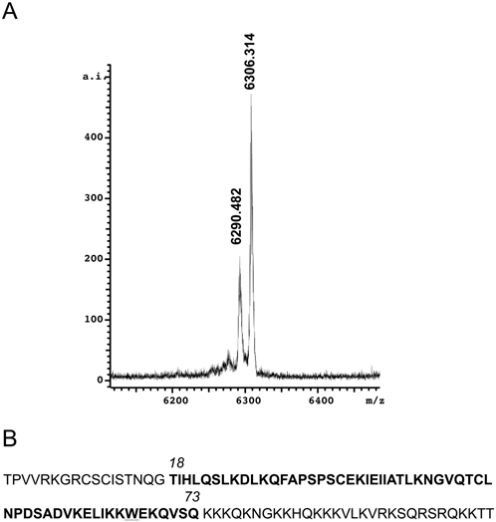
CXCL9 is trimmed amino-terminally and carboxy-terminally by SpeB. Panel A: SpeB-processed CXCL9 was analyzed by MALDI-TOF mass spectroscopy. Masses in Dalton are indicated above the to main intensity peaks. Panel B: The primary sequence of mature CXCL9 with the proposed SpeB-generated fragment constituting amino acids 18–73 in bold. The potentially oxidized tryptophan at position 67 is underlined.

The finding that SpeB-processed CXCL9 had retained antibacterial activity but lost its receptor stimulating activity was somewhat surprising. We therefore attempted to identify the SpeB-generated fragment of CXCL9 by mass spectrometry, after several failed attempts to obtain an NH_2_-terminal sequence by Edman degradation. CXCL9 was digested with SpeB as described above for the receptor activating experiments and analyzed using MALDI-TOF MS and matching against theoretical fragments of the known CXCL9 holopeptide. These experiments demonstrated two major CXCL9 cleavage products of 6,290.482 and 6,306.314 Dalton respectively (Fig. 4A). The Expasy FindPept tool analysis revealed that this corresponds to a peptide from CXCL9 spanning amino acids 18 to 73 with a mass error of 0.03 Da (Fig. 4B). Furthermore, the additional peak most likely represents the mass of the same peptide but with an oxidized tryptophan (Fig. 4B, W), strengthening the identification of the peptide within CXCL9. This suggests, that the proteolytic activity of SpeB on CXCL9 occurs both in the amino-terminal and carboxy-terminal part of the molecule, generating a fragment spanning the amino acids 18 to 73 of the CXCL9 holopeptide.

**Figure 5:**
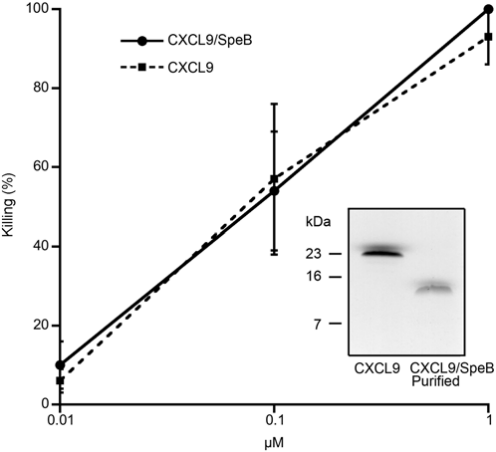
Dose dependent killing of *S. pyogenes* killing by CXCL9 and the SpeB generated fragment. The purified 18–73 SpeB generated CXCL9 fragment was compared to the intact CXCL9 for killing of *S. pyogenes* strain AP1. Curves represent mean bacterial killing in percent with standard error determined from at least three independent experiments. Inset: Intact CXCL9 and ion exchange chromatography purified SpeB generated CXCL9 fragment separated on 16.5% SDS-PAGE.

In order to confirm the antibacterial activity of the SpeB generated CXCL9 fragment, a peptide spanning amino acids 18 to 73 of CXCL9 was synthesized. This peptide had no receptor stimulating activity that was expected, but somewhat surprising the antibacterial activity was also very low (data not shown). This could be interpreted as a misidentification of the fragment or that the synthetic peptide did not have its functionally correct conformation. In order to elucidate this, we purified SpeB-processed CXCL9 using anion exchange chromatography. This approach is based on the fact that minor remaining fragments, especially from the carboxy-terminal end of CXCL9 are highly positively charged and would, under the conditions used, be filtrated through the column, while the 18–73 fragment with a lower pI will interact with the matrix (See Table 2). Using this method we could purify the SpeB generated CXCL9 fragment to homogeneity (Fig. 5, inset). This purified fragment was subsequently tested for antibacterial activity against *S. pyogenes* using the viable count assay as described above. This revealed that SpeB-generated MIG 18–73 has an antibacterial activity against *S. pyogenes* comparable to intact CXCL9 (Fig. 5).

**Table 2:** Biochemical properties of CXCL10, CXCL11 and CXCL9 (intact and SpeB cleaved).

Peptide	Molecular mass (Da)	pI	No. of hydrophobic residues	No. of hydrophilic residues	Positive residues	Negative residues
CXCL10	8645.4	11.22	31	23	17	6
CXCL11	8306.10	10.34	30	20	17	6
CXCL9	11723.38	10.59	29	38	29	7
CXCL9 18–73	6292.66	8.39	22	18	9	7

### Homology modeling of the SpeB generated CXCL9 fragment

**Figure 6:**
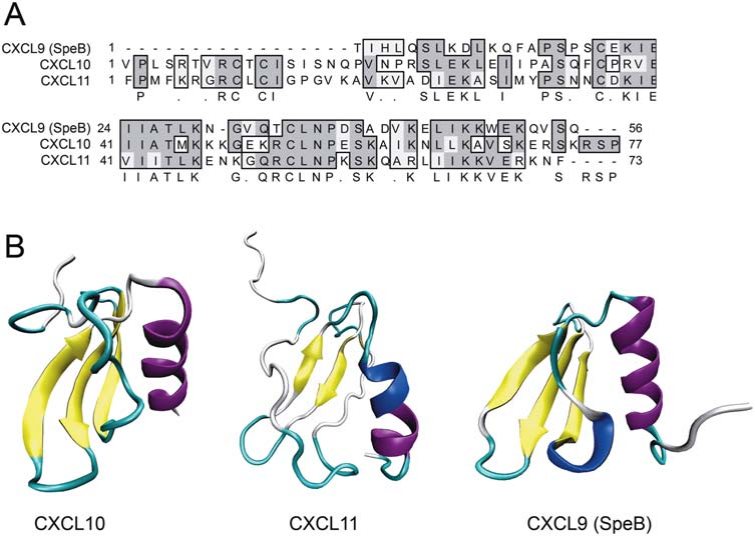
Sequence and structural comparison of CXCL10, CXCL11, and SpeB-processed CXCL9. Panel A: ClustalW alignment of the SpeB-generated fragment of CXCL9 (18–73) with mature CXCL10 and CXCL11. Amino acid identities are shown in dark grey, similarities in light grey, and consensus sequence is shown under the alignment. Panel B, visualization of the known structures of mature CXCL10 and CXCL11, and homology model of the SpeB-generated CXCL9 fragment (18–73) using a CXCL7 -variant as the template. ®-strands are shown in yellow, 〈-helices in purple, 3_10_-helices in blue, and loops in turquoise.

It was somewhat surprising that CXCL9 was much more resistant to SpeB compared to the closely related molecules CXCL10 and CXCL11. When aligning the amino acid sequences of SpeB-processed CXCL9 with CXCL10 and CXCL11, there are no obvious explanations for this phenomenon except minor stretches of divergent sequence (Fig. 6A). Therefore, we generated a structure homology model of SpeB-processed CXCL9 using the recently developed M4T method [44] with a variant form of CXCL5 [45] as template. This model was compared to the known structures of CXCL10 and CXCL11 [46], [47]. This revealed that SpeB-processed CXCL9 is strikingly similar to mature CXCL10 and CXCL11 with three amino-terminal antiparallel β-sheets and a carboxy-terminal α-helix (Fig. 6B). Some of the differences that can be noted is that CXCL11 has a somewhat less compact structure compared to both CXCL9 and CXCL10 with several internal flexible loops, and that CXCL has an additional internal predicted 3_10_-helix between the β-sheets and the carboxy-terminal α-helix.

## Discussion

For the first time we show that the cysteine proteinase SpeB from the important human pathogen *S. pyogenes* has differential proteolytic activity on human chemokines. Most chemokines are completely degraded, while a few are partly or completely resistant to the proteolytic activity of SpeB. The differential activity seen on the closely related ELR-negative CXC chemokines CXCL9, CXCL10, and CXCL11 was particularly interesting, since these chemokines are highly expressed by inflamed pharyngeal epithelium and have strong antibacterial activity against *S. pyogenes* [31]. More detailed analysis of these chemokines revealed that CXCL10 and CXCL11 were completely degraded by SpeB, resulting in loss of antibacterial activity and consequently also receptor stimulating activity. In contrast, CXCL9 has a SpeB-resistant core spanning amino acids 18–73 that has no receptor stimulating activity but with retained antibacterial activity against *S. pyogenes*. The finding that such a fragment of CXCL9 has no CXR3-stimulating activity is in concordance with previous findings that CXCL9 1–74 and 74–103 have low or no chemotactic activities [48], and further establishes that both the carboxy-terminal and amino-terminal parts of MIG are important for activation of the receptor. Our previous finding that the synthetic peptide CXCL9 57–83 has a strong antibacterial against *S. pyogenes* [31] could appear as being in conflict with our present finding that synthetic CXCL9 18–73 is not antibacterial and thereby indicate a misidentification of the SpeB generated fragment. In addition to what we consider unambiguous mass spectroscopy identification, CXCL9 57–83 is derived from the putative amphiphatic α-helix containing a classical sequence of antibacterial amino acids. CXCL9 18–73 on the other hand is derived from the NH_2_-terminal region of the holopeptide, and only contains some of the COOH-terminal antibacterial motifs. Native CXCL9 (and recombinant) is held together by two disulphide bonds corresponding to what in CXCL8 and CXCL10 have been shown to be crucial for oligomerization. The peptide CXCL9 18–73 is synthesized as a linear peptide and it is very unlikely that it can adopt a structure that allows the naturally occurring oligomerization. We hypothesize that CXCL9 18–73 fragments generated by SpeB oligomerize and thereby expose their antibacterial motifs in combination, enhancing the antibacterial activity.

The structural differences between the homology model of CXCL9 18–73 on one hand, and CXCL10 and CXCL11 on the other hand, might partly explain the SpeB resistance of this part of CXCL9; since SpeB is known to preferentially cleave extended loops [49]. Nevertheless, most of the explanation probably lies within the part of CXCL9 that is not in the model; the amino-terminal and carboxy-terminal degraded by SpeB cannot be modeled, indicating that these parts are largely unstructured or flexible. This is also supported by the fact that there currently is no experimentally determined structure of CXCL9. These potentially unstructured or flexible parts of CXCL9 might serve as “bait” for SpeB and other proteases to divert the activity from the antibacterial core of the molecule. From an evolutionary point of view, it is interesting that CXCL9 seems to have evolved towards a robust antibacterial chemokine with relatively weak receptor interaction, while CXCL10 and CXCL11 both have more efficient interaction with the receptor but are less antibacterial and more protease sensitive than CXCL9. What is very surprising is that the highly antibacterial 18–73 fragment of CXCL9 has a predicted structure that is very similar to CXCL10 and CXCL11, but is not as highly positively charged as these molecules (Fig 6B and Table 2). This might indicate that the overall structure of an chemokine is more important for antibacterial activity than overall charge, at least against *S. pyogenes* and possibly other related Gram-positive bacteria.

Taken together, or results indicate that SpeB has the capacity to destroy most of the signaling properties of chemokines expressed by an inflamed epithelium by degradation of CXCL10, CXCL11, and removing the signaling activity of CXCL9 through CXCR3. CXCL9 on the other hand seems to have evolved to resist the SpeB activity and to preserve its antibacterial activity towards *S. pyogenes*. In the context of a bacterial infection with release of several chemokines, other antimicrobial compounds, and importantly other bacterial proteins the situation is very complex and cannot be reduced to a single interaction between a protease and a substrate. For instance, the streptococcal inhibitor of complement (Sic) can probably inhibit the antibacterial activity of the SpeB generated CXCL9 fragment since it is known to inhibit intact CXCL9-11 [31]. Furthermore, the cell wall-anchored serine proteinase SpyCEP, most likely contributes to inhibition of pro-inflammatory and antibacterial activities of chemokines during infection [38], [39], [40], [41], [50]. Nevertheless, we believe that our results deepen the understanding of innate immunity control of *S. pyogenes* and emphasize the role for CXCL9 as one of the major antimicrobial compounds on inflamed airway epithelium.

## Materials and Methods

### Chemicals, bacterial strains and proteins

Recombinant human chemokines were purchased from Peprotech (London, UK). All other chemicals were purchased from Sigma (St Louis, MO) if not noted otherwise. The *S. pyogenes* strain AP1 (40/58) of serotype M1 from the WHO Collaborating Center for Reference and Research on Streptococci, Prague, Czech Republic. The *S. pyogenes* strain AL1 is an isogenic derivative of strain AP1 lacking active SpeB production generated as previously described [19], [51]. For maximal SpeB production, *S. pyogenes* was cultured in C-medium (CM) consisting of 0.5% (w/v) Proteose Peptone No. 2 (Difco, Detroit, MI) and 1.5% (w/v) yeast extract (Oxoid, Basingstoke, England) dissolved in CM buffer (10 mM K_2_PO_4_, 0.4 mM MgSO_4_, and 17 mM NaCl pH 7.5) [52]. When culturing strain AL1, CM was supplemented with 150 µg / ml of kanamycin. SpeB was purified from the culture supernatant of AP1 cultured in CM using ion exchange chromatography as previously described [53]. Activity of SpeB was determined by active site titration using the specific cysteine proteinase inhibitor L-*trans-*epoxysuccinylleucylamido(4-guanidino)butane (E-64) [43] as previously described [54]. Throughout the paper, the amount of SpeB is described as pmol active enzyme.

### Incubation of chemokines with SpeB or culture medium

Two µg each of recombinant chemokines CXCL9, CXCL10, ICXCL11, CXCL4, CXCL8, CXCL1, CXCL2, CXCL3, CXCL5, CXCL6, CXCL7, CXCL12, CXCL13, CXCL14, CXCL16, CCL5, CCL20, CCL28, XCL1, and CX3CL1 were incubated with 1 pmol SpeB in PBS supplemented with 10 mM dithiothreitol (DTT) for 2 h at 37°C. Two µg of recombinant CXCL9 was incubated for 2 h at 37°C with 5, 0.5, or 0.05 µl of 0.2 µm-filtered culture medium from AP1 or AL1 in PBS supplemented with 10 mM DTT. Samples were analyzed on 16.5% Tris-Tricine SDS-PAGE and stained with Commassie Brilliant Blue.

### Viable count assay

AP1 bacteria were cultured to mid-exponential growth phase in Todd-Hewitt Broth (TH) (Becton Dickinson, Sparks, MD), washed, and diluted in 10 mM Tris-HCl (pH 7.5) with 5 mM glucose. 50 µl of bacteria (2×10^6^ colony forming units (CFU) / mL) were incubated together with chemokines, at various concentrations or buffer alone for 2 h at 37°C. To quantitate bactericidal activity, serial dilutions of the incubation mixtures were plated on agar-solidified TH and the number of CFUs were determined after an overnight incubation at 37°C.

### CXCL9/SpeB incubation, CXCR3 transfectants, and calcium flux experiments

10 µg of CXCL9 was incubated with 5 pmol SpeB in 20 µl PBS supplemented with 10 mM DTT or in PBS/DTT alone for 2 h at 37°C. Reactions were terminated by addition of E-64 to a final concentration of 20 µM. Samples were analyzed on 16.5% Tris-Tricine SDS-PAGE. Stable transfectants of the pre-B cell line 300-19 expressing CXCR3 [28], were used to assay for receptor dependent responsiveness to CXCL9 and SpeB-processed CXCL9, respectively. Fura-2 loaded transfectants were stimulated using 100 nM intact CXCL9, CXCL9 incubated with SpeB / DTT / E-64 as described above, or SpeB / DTT / E-64. The [Ca^2+^]_i_ -related fluorescence changes were recorded as described [28], [55], [56].

### Negative staining and transmission electron microscopy

Bacteria were incubated for 2 h at 37°C in incubation buffer with the chemokines placed on a carbon coated copper grid and negatively stained as described by Engel and Furthmayr [21]. The samples were washed twice with water and stained on two drops 0.75% uranyl formate. Samples were observed using a Jeol JEM 1230 transmission electron microscope operated at 60 kV accelerating voltage, and recorded with a Gatan Multiscan 791 CCD camera.

### Statistical analysis

Statistical significance was determined based on the Student's *t*-test for paired observations.

### Determination of the CXCL9 cleavage sites by MALDI-TOF Mass Spectrometry (MS)

The CXCL9/SpeB reactions, terminated with E-64 as described above, were desalted and concentrated using C18 Ziptips (Millipore, Bedford, MA) according to the manufacturer's instructions. Briefly, the micro-columns were washed with 0.1% TFA and eluted with 1 µl 50–90% acetonitrile in 0.1% TFA directly onto MALDI Anchorchip target plates pre-spotted with 1 µl of 1 mg/ml sinnapinic acid (SA) in 90% acetonitrile / 0.1% TFA. The MALDI target plate was loaded into a Bruker Reflex III MALDI-TOF mass spectrometer (Bruker Daltonic GmbH, Bremen, Germany). The polarity of the instrument was set for positive ions with a delayed extraction and the detector for reflector mode. The acceleration voltage was 20 kV and 50–75 shots per sample were summed in each spectrum for an improved signal-to-noise ratio. Spectra were calibrated using insulin and myoglobin standards diluted to 1–2 µM and applied as above in 1 µl of 90% acetonitrile/0.1% TFA. Evaluated machine-specific protocols and settings for the mass spectrometer were used for each of the calibrants. The EXPASY server FindPept tool (http://www.expasy.ch) was used to search for experimentally obtained peptide masses matching with the CXCL9 sequence considering mass values as well as post-translational / experimental modifications of CXCL9. Tolerance was set to 0.5 Daltons, cysteines in reduced form and methionines as well as tryptophans as oxidized.

### Purification of the SpeB-generated CXCL9 fragment

For ion exchange purification of the SpeB generated CXCL9 fragments 200 µg of SpeB was incubated with 40 pmol SpeB and 10 mM DTT in 400 µl of PBS. The sample was applied to a HiTrap SP HP column (GE Healthcare, Uppsala, Sweden) using an Äkta Prime (GE Healthcare) liquid chromatography system. The column was washed with 5 column volumes of 50 mM HEPES (pH 7.5) and proteins were eluted using a linear gradient over 25 column volumes reaching 1 M NaCl. The fractions were analyzed on SDS-PAGE and relevant fractions were pooled and concentrated using a Centricon YM 3000 (Millipore).

### Homology modeling and structural comparisons

A homology model of the SpeB-processed form of CXCL9 was generated using Multiple Mapping Method with Multiple Templates (M4T) (http://www.fiserlab.org/servers/m4t) [44] using a NAP-2/CXCL7 variant (PDB 1TVX) [45] as a template. The CXCL9 model and the previously determined structures of CXCL10 (PDB 1LV9) [46] and CXCL11 (PDB 1RJT) [47] were visualized using VMD 1.8.6 (http://www.ks.uiuc.edu/Research/vmd/) [57] and high-resolution images were generated using the Tachyon ray tracer [58].
